# Increased visceral fat volume raises the risk for recurrence of hepatocellular carcinoma after curative treatment

**DOI:** 10.18632/oncotarget.24500

**Published:** 2018-02-16

**Authors:** Kenji Imai, Koji Takai, Toshihide Maeda, Satoshi Watanabe, Tatsunori Hanai, Atsushi Suetsugu, Makoto Shiraki, Masahito Shimizu

**Affiliations:** ^1^ Department of Gastroenterology/Internal Medicine, Gifu University Graduate School of Medicine, Gifu, Japan

**Keywords:** hepatocellular carcinoma, recurrence risk, visceral fat volume, obesity, metabolic disorder

## Abstract

Obesity is a risk factor for the development of hepatocellular carcinoma (HCC). This study aimed to assess the influence of visceral fat on the recurrence of HCC after curative treatment. In 207 curative cases of HCC, the cross-sectional areas of visceral and subcutaneous fat mass on the computed tomographic image at the fourth lumbar vertebra were normalized by the square of the height to obtain the visceral fat mass index (VFMI) and the subcutaneous fat mass index (SFMI), respectively. Whether VFMI and SFMI contributed to recurrence of HCC and overall survival was analyzed using a Cox proportional hazards model. Increased VFMI was significantly associated with recurrence of HCC (*P* = 0.006), whereas SFMI was not (*P* = 0.502). When the patients were divided based on the optimal cut off value for VFMI (47.2 cm^2^/m^2^), obtained from maximally selected rank statistics to best predict the risk for recurrence, the higher VFMI group (*n* = 79) had more probability of recurrence than the lower VFMI group (*n* = 128) (log rank test, *P* = 0.002). There were significant differences in body mass index (*P* < .0001), SFMI (*P* < .0001), L3 skeletal muscle index (*P* < .0001), platelet count (*P* = 0.003), hemoglobin A1c (*P* < .0001), triglycerides (*P* = 0.004), serum leptin (*P* = 0.043), and underlying liver disease (*P* < .0001) between the groups. Neither VFMI (*P* = 0.689) nor SFMI (*P* = 0.117) significantly contributed to overall survival. VFMI, which is involved in obesity and its related metabolic disorders such as diabetes, hyperlipidemia, and adipokine imbalance, is an extremely promising indicator that can predict the risk of recurrence of HCC after curative treatment.

## INTRODUCTION

Hepatocellular carcinoma (HCC) is one of the most common malignancies worldwide, accounting for approximately 500000 deaths annually [1]. HCC generally develops in patients with liver cirrhosis due to persistent hepatitis B virus (HBV) or hepatitis C virus (HCV) infection, alcohol consumption, and immune-related hepatitis [1]. The prognosis for patients with HCC is very poor because of its extremely higher recurrence rate; in fact, the 5-year recurrence rate after curative treatment is reported to be more than 70% [2, 3]. Therefore, development of effective surveillance strategies for HCC patients after curative treatment according to the predicted recurrence risk factors is essential to improve the prognosis of this malignancy.

Several factors, including male gender, presence of cirrhosis, high α-fetoprotein, large tumor foci, multiplicity of tumors, pathologically high-grade atypia of tumor cells, and presence of portal venous invasion, have been reported to raise the recurrence risk for HCC after curative treatment [4–7]. In addition, obesity and related metabolic disorders such as diabetes mellitus are also significantly involved in the development of HCC. A higher level of homeostasis model assessment-insulin resistance, an indicator of insulin resistance, was associated with increased risk of HCC recurrence after curative treatment [8]. Increases in the serum levels of leptin and oxidative stress, both of which are involved in obesity, also predicted the risk of HCC recurrence [9, 10]. These findings suggest that obesity, (*i.e.*, an excessive accumulation of fat) may promote liver carcinogenesis and, therefore, might be a candidate factor that would predict HCC recurrence after curative treatment.

Recent studies have revealed that differences in body composition may be critical determinants of prognosis in patients with HCC. For example, sarcopenia, which is characterized by the loss of skeletal muscle mass, is associated with a poor prognosis of HCC [11]. Visceral adiposity, which is significantly related to obesity and metabolic syndrome, is also an independent predictor of survival in HCC patients [12]. In addition, a large prospective multicenter cohort study in Europe showed a significant relationship between abdominal obesity and liver carcinogenesis [13]. A cross-sectional study has reported that visceral fat accumulation is implicated in the recurrence of HCC in patients with non-alcoholic steatohepatitis (NASH) [14]. The results of these reports indicate that visceral adiposity may have an impact on HCC development.

In this study, we measured the amounts of visceral and subcutaneous fat using computed tomography (CT) images and calculated the visceral fat mass index (VFMI) and subcutaneous fat mass index (SFMI) in HCC patients. The purpose of this study was to investigate whether these indices would affect the recurrence-free and overall survival in patients with HCC who received curative treatment.

## RESULTS

### Baseline characteristics and laboratory data of patients

The baseline characteristics and laboratory data of the 207 patients (145 males and 62 females; average age, 70.4 years) are shown in Table 1. The average body mass index (BMI) for enrolled patients was 23.1 kg/m^2^. Among these patients, 128 (61.8%) were infected with HCV and 23 (11.1%) with HBV, and the other etiologies included alcohol use in 9 (4.3%), primary biliary cholangitis in 2 (1.0%), and suspected NASH or unknown in 45 (21.7%). The average VFMI and SFMI were 40.5 and 37.4 cm^2^/m^2^, respectively. Female patients had a higher SFMI than male patients (51.8 vs. 31.2 cm^2^/m^2^, *P* <.0001), but the VFMI was not different between females and males (37.1 vs. 41.9 cm^2^/m^2^, *P* = 0.194).

**Table 1:** Baseline demographic and clinical characteristics

Variables	Total patients (*n* = 207)
Sex (male/female)	145/62
Age (years)	70.4 ± 9.3
Etiology (HBV/HCV/others)	23/128/56
BMI (kg/m^2^)	23.1 ± 3.2
L3 SMI (cm^2^/m^2^)	44.3 ± 8.1
VFMI (cm^2^/m^2^)	40.5 ± 24.7
SFMI (cm^2^/m^2^)	37.4 ± 21.1
Child-Pugh classification (A/B/C)	156/55/5
ALB (g/dL)	3.8 ± 0.5
ALT (IU/L)	40.8 ± 30.6
T-Bil (mg/dL)	1.0 ± 0.6
PLT (×10^4^/mL)	13.1 ± 6.4
PT (%)	86.3 ± 16.4
FPG (mg/dL)	111.1 ± 34.0
HbA_1c_ (%)	6.1 ± 1.2
TG (mg/dL)	101.9 ± 56.5
Leptin (ng/mL)	6.1 ± 4.8
adiponectin (μg/mL)	11.0 ± 7.4
Initial therapy (resection/RFA)	95/112
Supplementation with BCAA (yes/no)	70/137
AFP (ng/dL)	575 ± 2474
PIVKA-II (mAU/mL)	6534 ± 47143
Stage (I/II/III/IV)	75/95/34/3

Values are presented as average ± standard deviation. HBV, hepatitis B virus; HCV, hepatitis C virus; BMI, body mass index; L3 SMI, third lumbar vertebra skeletal muscle index; VFMI, visceral fat mass index; SFMI, subcutaneous fat mass index; ALT, alanine aminotransferase; T-Bil, total bilirubin; PLT, platelet count; PT, prothrombin time; FPG, fasting plasma glucose; RFA, radiofrequency ablation; BCAA, branched chain amino acids; AFP, alpha-fetoprotein; PIVKA-II, protein induced by vitamin K absence or antagonists-II.

**Figure 1:**
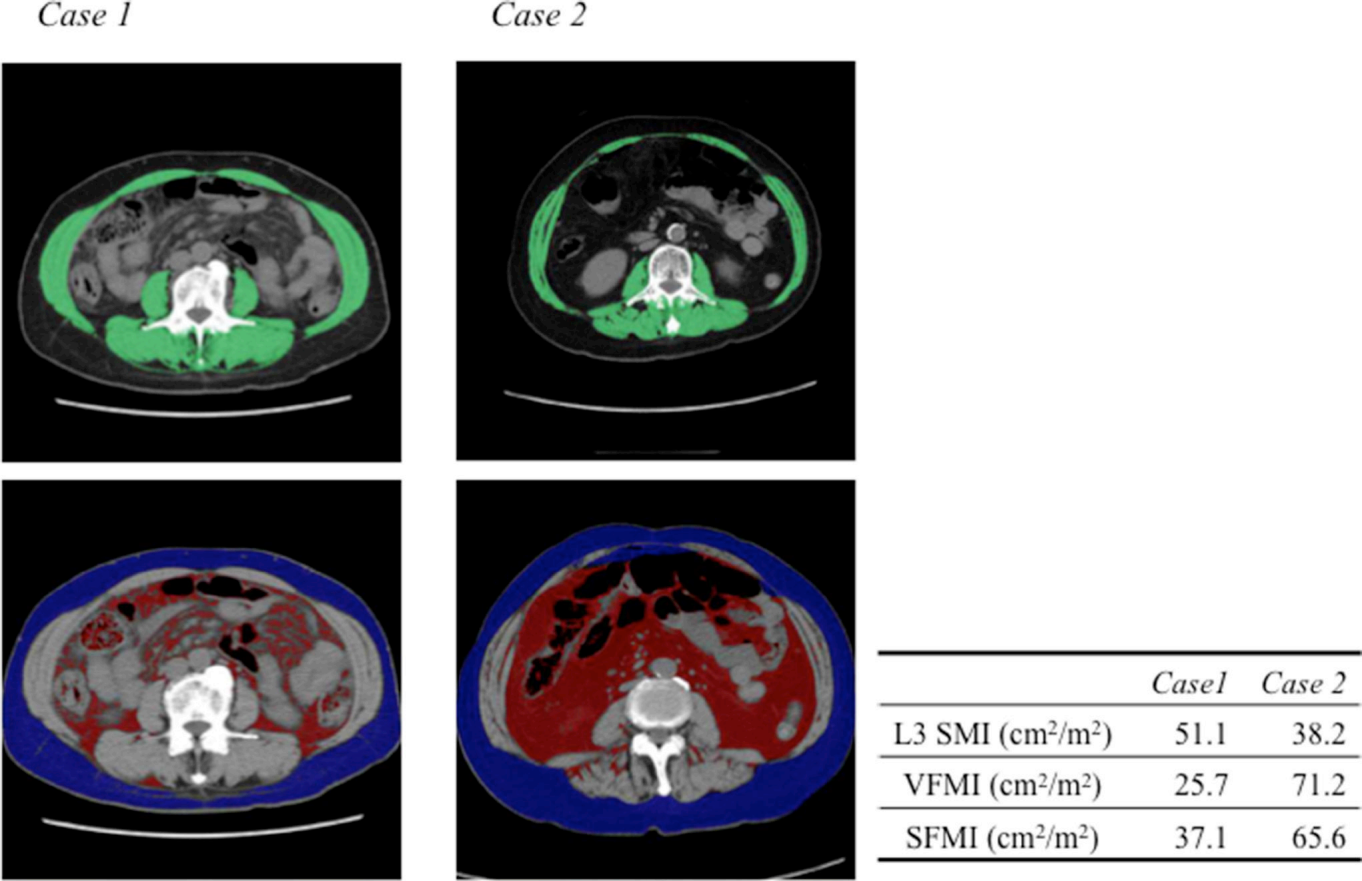
Computed tomographic images used for the assessment of L3 skeletal muscle index (SMI), visceral fat mass index (VFMI), and subcutaneous fat mass index (SFMI) Two cases of female HCC patients are shown. The body mass indices of the two patients were identical (25 kg/m^2^). Case 1 is a typical patient with higher SMI and lower VFMI and SFMI. Case 2 is a typical patient with lower L3 SMI and higher VFMI and SFMI. The green, red, and blue shading indicate skeletal muscle, visceral fat, and subcutaneous fat, respectively.

Examples of abdominal CT images from which L3 skeletal muscle index (L3 SMI), VFMI, and SFMI were obtained in two cases of female HCC patients with an identical BMI (25 kg/m^2^), but quite different body composition, are shown in Figure 1. Case 1 has an ideal body composition with higher skeletal muscle mass and lower visceral fat. On the other hand, case 2 has an utterly inverted body composition showing lower skeletal muscle mass and higher visceral fat. Importantly, these differences of body composition were not identifiable using BMI alone.

### Impact of VFMI and SFMI on overall survival and recurrence-free survival in patients with HCC after curative treatment

**Figure 2:**
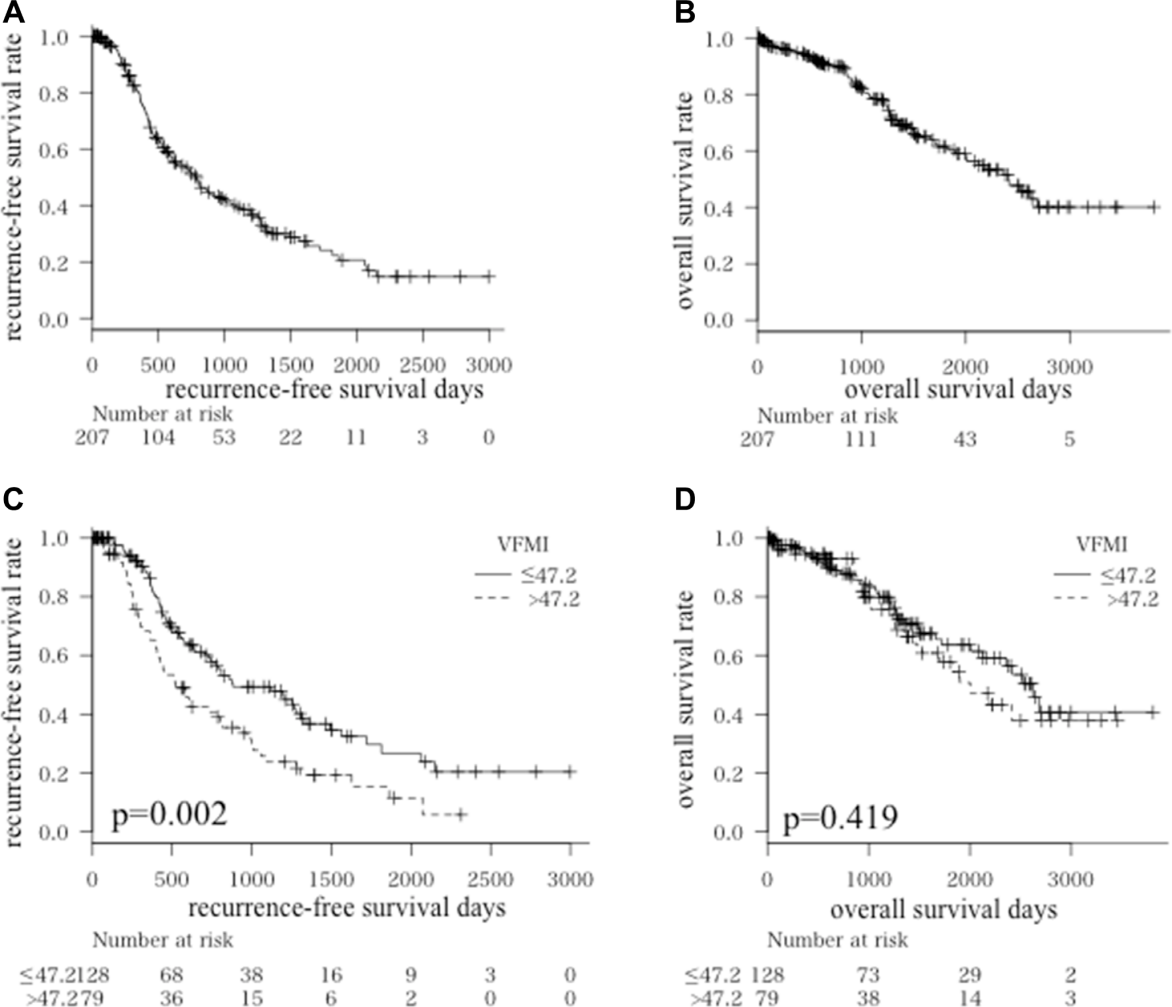
Kaplan-Meier curves for recurrence-free survival time (**A**) and overall survival time (**B**) in all patients Kaplan-Meier curves for recurrence-free survival time (**C**) and overall survival time (**D**) divided into higher visceral fat mass index (VFMI; > 47.2 cm^2^/m^2^) and lower VFMI (≤ 47.2 cm^2^/m^2^) groups.

The 1-, 3-, and 5-year recurrence-free survival rates of all enrolled patients were 77.1, 39.3, and 22.4%, respectively (Figure 2A), and the 1-, 3-, and 5-year overall survival rates were 94.7, 79.1, and 61.5%, respectively (Figure 2B). The Cox proportional hazards model showed that VFMI and SFMI did not affect overall survival (Table 2). On the other hand, VFMI affected recurrence-free survival [hazard ratio (HR): 1.010, 95% confidence interval (CI): 1.003–1.017, *P* = 0.006], whereas SFMI did not (HR: 1.003, 95% CI: 0.994–1.012, *P* = 0.502; Table 3).

**Table 2:** Cox proportional hazards model of whether VFMI and SFMI affected overall survival

Variables		95% CI		
	HR	lower	upper	*P* value
VFMI (cm^2^/m^2^)	0.999	0.992	1.005	0.689
SFMI (cm^2^/m^2^)	0.994	0.986	1.002	0.117

CI, confidence interval; HR, hazard ratio; VFMI, visceral fat mass index; SFMI, subcutaneous fat mass index.

**Table 3:** Cox proportional hazards model of whether VFMI and SFMI affected recurrence-free survival

Variable		95% CI		
	HR	lower	upper	*P* value
VFMI (cm^2^/m^2^)	1.010	1.003	1.017	0.006
SFMI (cm^2^/m^2^)	1.003	0.994	1.012	0.502

CI, confidence interval; HR, hazard ratio; VFMI, visceral fat mass index; SFMI, subcutaneous fat mass index.

We next set the most optimal cut-offs for VFMI at 47.2 cm^2^/m^2^ using maximally selected rank statistics and investigated the impact of VFMI on HCC recurrence and overall survival. As shown in Figure 2C and 2D, the higher VFMI group (> 47.2 cm^2^/m^2^) had HCC recurrences earlier compared to the lower VFMI group (≤ 47.2 cm^2^/m^2^, *P* = 0.002). However, there was no significant difference in the overall survival rate in these two groups (*P* = 0.419). These results indicate that a higher VFMI is associated with recurrence of HCC; however, this might not worsen the prognosis of patients with this malignancy.

**Figure 3:**
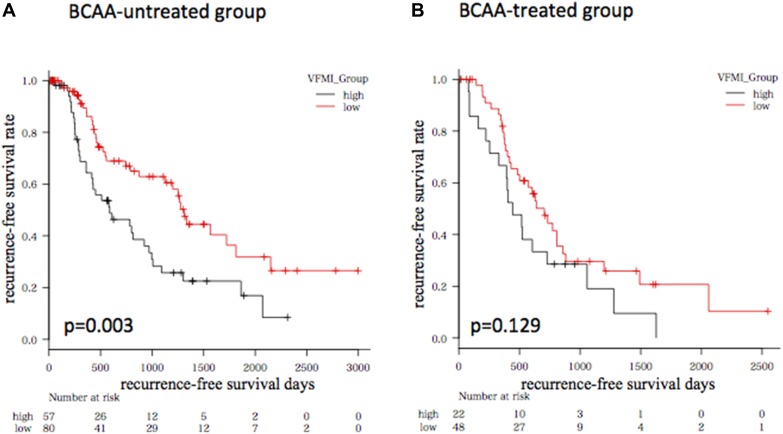
Kaplan-Meier curves for recurrence-free survival time in patients without (**A**) or with (**B**) BCAA supplementation. Patients were divided into higher visceral fat mass index (VFMI; > 47.2 cm^2^/m^2^) and lower VFMI (≤ 47.2 cm^2^/m^2^) groups, respectively.

We then evaluated the impact of branched chain amino acids (BCAA) supplementation on HCC recurrence because this agent was reported to suppress HCC development in liver cirrhotic patients with obesity [15]. In the present study, 70 cases used BCAA and they showed poor liver functional reserve, *e.g.*, the average serum albumin level was significantly low in BCAA-treated group (3.4 g/dL) compared to untreated group (3.9 g/dL, *P* <.0001). In BCAA-untreated group, the recurrence-free survival rate of the higher VFMI group was significantly low compared to the lower VFMI group (*P* = 0.003, Figure 3A). However, no significant difference was observed in the BCAA-treated group (*P* = 0.129, Figure 3B).

Table 4 shows the clinical characteristics and laboratory data of the higher (*n* = 79) and lower (*n* = 128) VFMI groups. There were significant differences in etiology (HBV/HCV/others = 6/36/37 vs. 17/92/19, *P <*.0001), BMI (25.3 vs. 21.7 kg/m^2^, *P <*.0001), L3 SMI (47.0 vs. 42.7 cm^2^/m^2^, *P <*.0001), VFMI (66.5 vs. 24.4 cm^2^/m^2^, *P <*.0001), SFMI (46.3 vs. 31.9 cm^2^/m^2^, *P <*.0001), platelet count (14.8 vs. 12.1 × 10^4^/mL, *P* = 0.003), hemoglobin A1c (6.5 vs. 5.9%, *P <*.0001), triglycerides (117.4 vs. 91.6 mg/dL, *P* = 0.004), and leptin (8.2 vs. 5.1 ng/mL, *P* = 0.043) between the groups, and most of these differences were associated with obesity and metabolic disorders.

**Table 4:** Baseline demographic and clinical characteristics of higher (> 47.2 [cm^2^/m^2^]) and lower (≤ 47.2 [cm^2^/m^2^]) VFMI groups

Variables	higher VFMI (*n* = 79)	lower VFMI (*n* = 128)	*P* value
Sex (male/female)	60/19	85/43	0.162
Age (years)	71.9 *±* 9.0	69.4 ± 9.4	0.060
Etiology (B/C/others)	6/36/37	17/92/19	< .0001
BMI (kg/m^2^)	25.3 *±* 2.9	21.7 ± 2.5	< .0001
L3 SMI (cm^2^/m^2^)	47.0 *±* 8.6	42.7 ± 7.4	< .0001
VFMI (cm^2^/m^2^)	66.5 *±* 15.2	24.4 ± 13.0	< .0001
SFMI (cm^2^/m^2^)	46.3 *±* 20.4	31.9 ± 19.6	< .0001
Child-Pugh classification (A/B/C)	70/27/2	86/28/3	0.298
ALB (g/dL)	3.9 *±* 0.50	3.7 ± 0.5	0.077
ALT (IU/L)	39.5 *±* 24.5	41.6 ± 33.9	0.625
T-Bil (mg/dL)	1.0 *±* 0.4	1.1 ± 0.7	0.198
PLT (×10^4^/μL)	14.8 *±* 6.9	12.1 ± 5.8	0.003
PT (%)	88.4 *±* 18.6	85.0 ± 14.8	0.154
FPG (mg/dL)	116.2 *±* 35.6	108.0 ± 32.7	0.094
HbA_1c_ (%)	6.5 *±* 1.2	5.9 ± 1.2	< .0001
TG (mg/dL)	117.4 *±* 51.7	91.6 ± 57.5	0.004
Leptin (ng/mL)	8.2 *±* 6.3	5.1 ± 3.7	0.043
adiponectin (μg/mL)	8.6 *±* 8.1	12.1 ± 7.0	0.155
Initial therapy (resection/RFA)	43/36	52/76	0.062
Supplementation with BCAA (yes/no)	57/22	80/48	0.175
AFP (ng/dL)	742 *±* 3394	473 ± 1705	0.452
PIVKA-II (mAU/mL)	9739 *±* 64841	4616 ± 32412	0.455
Stage (I/II/III/IV)	25/39/14/1	50/56/20/2	0.771

Values are presented as average ± standard deviation. VFMI, visceral fat mass index; HBV, hepatitis B virus; HCV, hepatitis C virus; BMI, body mass index; L3 SMI, third lumbar vertebra skeletal muscle index; SFMI, subcutaneous fat mass index; ALT, alanine aminotransferase; T-Bil, total bilirubin; PLT, platelet count; PT, prothrombin time; FPG, fasting plasma glucose; RFA, radiofrequency ablation; BCAA, branched chain amino acids; AFP, alpha-fetoprotein; PIVKA-II, protein induced by vitamin K absence or antagonists-II.

## DISCUSSION

A large number of studies have demonstrated the association between obesity and development of HCC [16, 17]. Hepatic manifestations of obesity and the metabolic syndrome are collectively termed nonalcoholic fatty liver disease, approximately 20% of which cases present as NASH where cirrhosis and HCC can emerge [18]. Obesity also enhances the effect of other established risk factors of HCC such as alcohol cirrhosis and viral hepatitis [19, 20]. Since obesity has become more prevalent worldwide, it is significantly important to be able to predict obesity-related HCC and to develop suitable surveillance strategies for this malignancy in obese patients. The results of the present study clearly showed the first evidence that visceral adiposity, but not subcutaneous adiposity, is a predictor of recurrence of HCC after curative treatment. These findings, which are consistent with those of a previous study investigating NASH patients [14], may suggest that evaluation of visceral fat volume is useful to identify which HCC patients are prone to recurrence. It should be noted that about 80% of cases were NASH-unrelated HCC in the present study. Our finding may indicate that visceral fat accumulation increases the risk of HCC recurrence regardless of etiology.

Several pathophysiological mechanisms linking visceral adiposity and liver carcinogenesis have been reported, including adipose tissue remodeling and pro-inflammatory adipokine secretion [21, 22], ectopic lipid accumulation and lipotoxicity [23], and the growth effects of insulin and insulin-like growth factors [24, 25]. Amounts of adipose tissue causing inflammation play a critical role in liver tumorigenesis, which suggesting that the tissue might be a possible target for preventing obesity-related HCC [26–28]. An increased level of oxidative stress, which contributes to liver carcinogenesis [9], is also associated with visceral fat accumulation [18, 23]. Therefore, visceral adipose tissue, but not subcutaneous adipose tissue, is regarded as an organ that can modulate a range of systemic effects. In the present study, insulin resistance and increased levels of serum leptin, both of which are involved in the recurrence of HCC [8, 10], were observed in the patients with higher VFMI. These results may indicate that accumulation of visceral fat increases the risk of HCC recurrence, at least in part, by inducing insulin resistance and hyperleptinemia.

Evaluation of body composition is important to determine the prognosis in patients with HCC. Interestingly, the higher VFMI group (*i.e.*, the early recurrence group) in this study showed a higher L3 SMI, which is a good prognostic factor in HCC [11, 29]. Moreover, VFMI was also significantly correlated with L3 SMI (Pearson's correlation coefficient: 0.276, *P* < .0001, data not shown). Since the patients with a higher VFMI maintained skeletal muscle volume, the overall survival might not be shortened in these patients even though they are at a high risk for recurrence of HCC. Several studies have revealed that visceral adiposity independently predicts mortality in patients with HCC [12, 30]. On the other hand, it is also reported that high visceral fat amount is involved in high BCAA level, a key molecules which can suppress obesity-related liver carcinogenesis [15, 26, 31], and improved overall survival in HCC patients [32]. Decreasing serum BCAA levels is also associated with sarcopenia [33]. Therefore, it might be important to assess body composition and, probably, serum BCAA level to predict the recurrence risk of HCC [34].

The BMI is simple to use, but is a limited anthropometric index because it cannot assess regional fat distribution or skeletal muscle volume. Accumulation of visceral fat is associated with increased level of hepatic inflammatory activity, which stimulates liver carcinogenesis, in HCV-positive patients, whereas BMI is not correlated with this activity [35]. Therefore, estimation of VFMI and L3 SMI, both of which are determined by CT images performed in the clinical setting for HCC, is beneficial to predict the risk of HCC recurrence. In particular, patients such as the aforementioned case 2 (Figure 1), who showed higher VFMI with lower L3 SMI, may require careful surveillance for recurrence of HCC. It should be noted that sarcopenic obesity is associated with poor survival in patients with HCC [11].

A question posed by the findings of the present study is whether targeting visceral adiposity might prevent the development and recurrence of HCC, especially in obese patients. It is also presumed that reduction of visceral fat volume while maintaining skeletal muscle volume may improve the prognosis of HCC patients. To achieve this purpose, exercise and nutrition therapies are the most promising interventions. Exercise therapy can attenuate inflammation, oxidative stress, and insulin resistance, all of which are not only involved in obesity-associated hepatocarcinogenesis [18], but also cause sarcopenia [36]. Nutrition therapy is also effective in improving visceral adiposity, as there is a strong association between energy intake and visceral fat accumulation [18, 21, 23]. Supplementation with BCAA, which is one of the main agents of nutrition therapy in cirrhotic patients [35, 37, 38], is promising because it can suppress the development of HCC in cirrhotic obese patients [15]. BCAA supplementation also inhibits obesity-related liver carcinogenesis in mice by attenuating inflammation in white adipose tissue [26], which suggests that BCAA may play a key role in the prevention of HCC development and recurrence in obese patients. Interestingly, higher VFMI did not have an effect on HCC recurrence when the patients were supplemented with BCAA, whereas BCAA-untreated patients having higher VFMI showed high risk for recurrence in the present study.

One of the limitations of the present study is that this is a retrospective study enrolling a small number of patients. Prospective studies in a larger number of patients must be undertaken to confirm the relationship between visceral adiposity and HCC recurrence in the future. In addition, prospective studies also should be performed which examine the preventive effects of BCAA supplementation on HCC recurrence in cirrhotic patients with higher VFMI.

In conclusion, HCC patients with a higher VFMI, an indicator of visceral fat mass presented in this study for the first time, are prone to recurrence. Higher VFMI also strongly reflects obesity and related metabolic disorders such as diabetes, hyperlipidemia, and hyperleptinemia. Therefore, VFMI is an extremely useful tool to recognize the complications of obesity and to predict the recurrence risk of HCC. Reduction of visceral fat volume while maintaining skeletal muscle volume might be an effective strategy to improve the prognosis of patients with HCC.

## MATERIALS AND METHODS

### Patients, treatment, and determination of recurrence

A total of 385 HCC patients were treated in our hospital between May 2006 and December 2016. Among them, 207 patients who received curative treatment, including surgical resection or radiofrequency ablation, were enrolled in this study. All patients were followed on an outpatient basis and had dynamic CT, magnetic resonance imaging (MRI), or ultrasound every 3 months after the initial treatment. Recurrent HCC was diagnosed when the typical findings of HCC were observed in segments different from where the initial lesions arose. Recurrence-free survival time was defined as the interval from the date of the initial treatment to the date of the recurrence or December 2016 for recurrence-free survivors.

HCC nodules were detected using imaging modalities, including dynamic CT, dynamic MRI, and abdominal arteriography. HCC was diagnosed based on a typical hypervascular tumor stain on angiography and typical dynamic study findings of enhanced staining in the early phase and attenuation in the delayed phase.

All study participants provided verbal informed consent, which was considered sufficient as this study followed an observational research design that did not require new human specimens, and instead relied only on preexisting samples. The study design, including this consent procedure, was approved by the ethics committee of the Gifu University School of Medicine on June 7, 2017.

### Image analysis of skeletal muscle and visceral and subcutaneous fat volumes

We measured skeletal muscle and visceral and subcutaneous fat volumes from a CT image (that had been taken solely for the purpose of diagnosing HCC prior to the initial treatment) using SYNAPSE VINCENT software (version 3.0, Fujifilm Medical, Tokyo, Japan), which enables specific tissue demarcation using Hounsfield unit thresholds. As an indicator of skeletal muscle mass, we used the L3 skeletal muscle index (L3 SMI, cm^2^/m^2^) obtained by normalizing the cross-sectional areas of the muscle (cm^2^) at the third lumbar vertebra on the CT image by the square of the height (m^2^). The L3 SMI is a well-known indicator of skeletal muscle volume and a demonstrated prognostic factor in HCC [11, 29]. In the same manner, the cross-sectional areas of visceral and subcutaneous fat mass (cm^2^) at the fourth lumbar vertebra (where solid organs such as the liver, kidneys, or spleen are not apparent) were measured using the built-in function in the SYNAPSE VINCENT software. These values were then normalized by the square of the height (m^2^) to obtain the VFMI (cm^2^/m^2^) and SFMI (cm^2^/m^2^), respectively.

### Statistical analysis

Baseline characteristics were compared using the Student's *t* test for continuous variables or the χ^2^ test for categorical variables. The Cox proportional hazards model was used to analyze whether VFMI and SFMI affected overall and recurrence-free survival. Overall survival and recurrence-free survival were estimated using the Kaplan-Meier method, and differences between curves were evaluated using the log-rank test. Maximally selected rank statistics were used to determine the most optimal cut-off so that the separation of the curves in the two groups was maximized [39]. Statistical significance was defined as *P* < 0.05. All statistical analyses were performed using R v3.3.1 (The R Project for Statistical Computing, Vienna, Austria; http://www.R-project.org/).

## Abbreviations

HCC: hepatocellular carcinoma
VFMI: visceral fat mass index
SFMI: subcutaneous fat mass index
HBV: hepatitis B virus
HCV: hepatitis C virus
NASH: non-alcoholic steatohepatitis
CT: computed tomography
BMI: body mass index
HR: hazard ratio
CI: confidence interval
L3 SMI: L3 skeletal muscle index
BCAA: branched chain amino acids
MRI: magnetic resonance imaging.
